# Severe *ACTA1*-related nemaline myopathy: intranuclear rods, cytoplasmic bodies, and enlarged perinuclear space as characteristic pathological features on muscle biopsies

**DOI:** 10.1186/s40478-022-01400-0

**Published:** 2022-07-09

**Authors:** Clémence Labasse, Guy Brochier, Ana-Lia Taratuto, Bruno Cadot, John Rendu, Soledad Monges, Valérie Biancalana, Susana Quijano-Roy, Mai Thao Bui, Anaïs Chanut, Angéline Madelaine, Emmanuelle Lacène, Maud Beuvin, Helge Amthor, Laurent Servais, Yvan de Feraudy, Marcela Erro, Maria Saccoliti, Osorio Abath Neto, Julien Fauré, Béatrice Lannes, Vincent Laugel, Sandra Coppens, Fabiana Lubieniecki, Ana Buj Bello, Nigel Laing, Teresinha Evangelista, Jocelyn Laporte, Johann Böhm, Norma B. Romero

**Affiliations:** 1 Myology Institute, Neuromuscular Morphology Unit, Reference Center of Neuromuscular Diseases Nord-Est-IDF, GHU Pitié-Salpêtrière, Paris, France; 2 Neuropathology and Neuromuscular Diseases Laboratory, Buenos Aires, Argentina; 3 Sorbonne Université, INSERM, Center for Research in Myology, Myology Institute, APHP, GHU Pitié-Salpêtrière, Paris, France; 4 Laboratoire de Biochimie Et Génétique Moléculaire, Pôle de Biologie, CHU Grenoble Alpes, Grenoble, France; 5 Université Grenoble Alpes, Inserm, U1216, CHU Grenoble Alpes, Grenoble Institut Neurosciences, Grenoble, France; 6 Servucio de Neurología Et Neuropatología, Hospital de Pediatría J.P. Garrahan, Buenos Aires, Argentina; 7 Institut de Génétique Et de Biologie Moléculaire Et Cellulaire (IGBMC), Inserm U 1258, CNRS UMR 7104, Université de Strasbourg, Illkirch, France; 8 Laboratoire de Diagnostic Génétique, Faculté de Médecine, CHRU, Strasbourg, France; 9 APHP Université Paris-Saclay, Pediatric Neuromuscular Unit, Hôpital Universitaire Raymond-Poincaré, Université de Versailles Saint-Quentin-en-Yvelines, Garches, France; 10 Centre de Références Des Maladies Neuromusculaires, Department of Paediatrics, University Hospital Liège & University of Liège, Liège, Belgium; 11 Department of Paediatrics, MDUK Oxford Neuromuscular Centre, University of Oxford, Oxford, UK; 12 Department of Neuropediatrics, Strasbourg University Hospital, Strasbourg, France; 13 Gutierrez Pediatric Hospital, Buenos Aires, Argentina; 14 Department of Pathology, Strasbourg University Hospital, Strasbourg, France; 15 Center of Human Genetics, Université Libre de Bruxelles, Brussels, Belgium; 16 Université Paris-Saclay, Integrare Research Unit UMR S951, Inserm, Evry, France; 17 Généthon, Université Evry, Evry, France; 18 Centre for Medical Research, University of Western Australia, Harry Perkins Institute of Medical Research, Perth, Australia

**Keywords:** *ACTA1*, Congenital myopathy, Nemaline rods, Intranuclear rods, Cytoplasmic bodies, Nuclear envelope, Neuromuscular junction

## Abstract

Nemaline myopathy (NM) is a muscle disorder with broad clinical and genetic heterogeneity. The clinical presentation of affected individuals ranges from severe perinatal muscle weakness to milder childhood-onset forms, and the disease course and prognosis depends on the gene and mutation type. To date, 14 causative genes have been identified, and *ACTA1* accounts for more than half of the severe NM cases. *ACTA1* encodes α-actin, one of the principal components of the contractile units in skeletal muscle. We established a homogenous cohort of ten unreported families with severe NM, and we provide clinical, genetic, histological, and ultrastructural data. The patients manifested antenatal or neonatal muscle weakness requiring permanent respiratory assistance, and most deceased within the first months of life. DNA sequencing identified known or novel *ACTA1* mutations in all. Morphological analyses of the muscle biopsy specimens showed characteristic features of NM histopathology including cytoplasmic and intranuclear rods, cytoplasmic bodies, and major myofibrillar disorganization. We also detected structural anomalies of the perinuclear space, emphasizing a physiological contribution of skeletal muscle α-actin to nuclear shape. In-depth investigations of the nuclei confirmed an abnormal localization of lamin A/C, Nesprin-1, and Nesprin-2, forming the main constituents of the nuclear lamina and the LINC complex and ensuring nuclear envelope integrity. To validate the relevance of our findings, we examined muscle samples from three previously reported *ACTA1* cases, and we identified the same set of structural aberrations. Moreover, we measured an increased expression of cardiac α-actin in the muscle samples from the patients with longer lifespan, indicating a potential compensatory effect. Overall, this study expands the genetic and morphological spectrum of severe *ACTA1*-related nemaline myopathy, improves molecular diagnosis, highlights the enlargement of the perinuclear space as an ultrastructural hallmark, and indicates a potential genotype/phenotype correlation.

**Supplementary Information:**

The online version contains supplementary material available at 10.1186/s40478-022-01400-0.

## Introduction

Nemaline myopathies (NM) form a genetically and clinically heterogeneous group of congenital myopathies characterised by early-onset hypotonia, muscle and facial weakness, respiratory distress, swallowing difficulties, delayed motor milestones, and skeletal deformities [20, 23]. To date, 14 causative NM genes have been identified (*ACTA1*, *ADSSL1*, *CFL2*, *KBTBD13*, *KLHL40*, *KLHL41*, *LMOD3*, *MYO18B*, *MYPN*, *NEB, TNNT1*, *TNNT3*, *TPM2*, *TPM3*) [20, 32, 34], and they primarily code for components of the contractile unit, the sarcomere, or for auxiliary proteins regulating sarcomeric function, stability, or turnover [13]. As a direct consequence of pathogenic mutations in the NM genes, muscle biopsy specimens from affected individuals typically show abnormal accumulations of sarcomeric structures known as nemaline rods.

Mutations in *ACTA1* account for more than half of the genetically characterised cases with severe NM. Most are heterozygous missense mutations occurring de novo and affecting highly conserved amino acids, while 10% are reported with autosomal recessive inheritance [17, 23]. *ACTA1* encodes skeletal muscle α-actin (αskm-actin), a multi-functional protein able to polymerize and form the helical strands of the thin filaments. The formation of dynamic cross-bridges with the thick myosin filaments causes a sliding movement between actin and myosin, resulting in the shortening of the contractile unit, and the generation of force to enable motion [7]. Besides classical nemaline rods, histological investigations of muscle sections from *ACTA1* patients often reveal other structural anomalies including focal disorganization (core-like areas) with or without inclusion of rods, congenital fibre type disproportion (CFTD), and—more rarely—intranuclear rods or zebra bodies [20, 30]. The coexistence of several anomalies on the same muscle biopsy specimen is frequently observed, indicating that the *ACTA1* mutations can variably impact-on αskm-actin structure, function, and positioning, and thereby interfere with normal muscle architecture and physiology at different levels.

Here, we provide the clinical description of a series of ten unreported patients with severe NM together with a thorough analysis of the muscle morphology and ultrastructure. Molecular investigations detected known and novel *ACTA1* mutations in all affected individuals, including nine heterozygous missense mutations and a single heterozygous stop-loss mutation affecting the original termination codon and predicted to result in an extended protein. Histological and ultrastructural investigations of the muscle biopsy specimens uncovered common *ACTA1*-related anomalies such as clusters of rods, intranuclear rods and cytoplasmic bodies, and we detected additional structure abnormalities affecting the perinuclear space and the neuromuscular junction.

## Patients and methods

### Cohort description

We established a cohort of ten patients (cases 1–10) with a severe form of early-onset myopathy, and we examined muscle histology and ultrastructure, and performed genetic testing (Table 1). We subsequently included three previously reported cases (11–13) with concordant molecular diagnosis and similar clinical and histopathological presentation. The following points were investigated and compared in the eight females and five males: birth weight and gestational age, antenatal and neonatal signs, respiration, orthopaedic and dysmorphic abnormalities, disease course, additional clinical features, and muscle morphology. Sample collection was performed for diagnostic purposes and with written informed consent from the legal guardians of the patients according to the declaration of Helsinki and its later amendments.

**Table 1:** Description of the clinical, morphological and genetics findings

Patient (sex)	Age at muscle biopsy	Birth weight	*ACTA1* mutations (bold = homozygous)	Ante/neonatal signs	Permanent Respiratory assistance	Disease course	Additional signs	Muscle histoenzymology	Electron microscopy	Reference Patients
1 (F)	2.5 m	3170 g (40 wGA)	c.109G > C (p.Val37Leu) Exon 2	Hypotonia, respiratory distress, difficulties sucking & swallowing	Yes	Deceased at 5 m	High-arched palate	Fiber size variability, type I fiber predominance, cytoplasmic rods	Myofibrillar disorganization, rods of variable size, mini-rods emanating from enlarged Z-line segments**, enlarged perinuclear space**, prominent heterochromatin	This report
2 (F)	3 m	1240 g (27 wGA)	c.113G > A (p.Gly38Asp) Exon 2	Hypotonia, hypomotility, respiratory distress, difficulties swallowing	Yes	Deceased at 3 m	Facial dysmorphy, low-set ears, high-arched palate, arthrogryposis, clubfeet, short ribs	Fiber size variability, type I fiber predominance, endomysial fibrosis, cytoplasmic rods	Myofibrillar disorganization, rods of variable size with filamentous protrusions, **cytoplasmic bodies**	This report
3 (M)	2 m	3400 g (38 wGA)	c.203C > A (p.Thr68Asn) Exon 3	Hypotonia, respiratory distress, difficulties sucking & swallowing	Yes	Alive at 4 y, permanent respiratory assistance, never walked	Elongated face, high-arched palate, arthrogryposis, pectus excavatum	Fiber size variability, type I fiber predominance, cytoplasmic rods	Myofibrillar disorganization rods of variable size	This report
4 (F)	1.5 m	Nd (39 wGA)	c.282C > A (p.Asn94Lys) Exon 3	Hypotonia	Yes	Deceased at 7w	Arthrogryposis	Fiber size variability, atrophy, cytoplasmic bodies, endomysial fibrosis	Myofibrillar disorganization, Mini-rods in filamentary areas, **cytoplasmic bodies, enlarged perinuclear space**	This report
5 (M)	39d + 12 m	3100 g (38 wGA)	c.283G > A (p.Glu95Lys) Exon 3	Hydramnios, reduced fetal movements, hypotonia, facial weakness, respiratory distress, difficulties sucking & swallowing	Yes	Deceased at 18 m	Elongated and hypo-mimic face, dropped-head syndrome, scoliosis; dysautonomia	Fiber size variability, type I fiber atrophy, cytoplasmic bodies, endomysial fibrosis	Myofibrillar disorganization, mini-rods in filamentary areas, **intranuclear rods, cytoplasmic bodies, enlarged perinuclear space, abnormal NMJ**	This report
6 (F)	6d	Nd	c.355G > C (p.Glu119Gln) Exon 3	Hypotonia, respiratory distress, difficulties swallowing	Yes	Deceased at 1 m	Arthrogryposis, right clubfoot	Fiber size variability, type I fiber predominance, type I fiber atrophy, cytoplasmic & intranuclear rods	Myofibrillar disorganization, rods of variable size with filamentous protrusions, **intranuclear rods, enlarged perinuclear space**	This report
7 (F)	3 m	2900 g (38 wGA)	c.493G > T (p.Val165Leu) Exon 4	Hypotonia, difficulties sucking & swallowing	Yes	Deceased at 2y7m	-	Fiber size variability, atrophy, intranuclear rods, endomysial fibrosis	Myofibrillar disorganization, cytoplasmic mini-rods with filamentous protrusions, **intranuclear rods, enlarged perinuclear space**	This report
8 (F)	21d	3000 g (40 wGA)	c.592C > T (p.Arg198Cys) Exon 4	Hypotonia, respiratory distress, difficulties sucking & swallowing	Yes	Deceased at 5y	Facial diplegia, high-arched palate, arthrogryposis, hip retractions	Fibre size variability, type I fiber predominance, atrophy, cytoplasmic & intranuclear rods, cytoplasmic bodies	Myofibrillar disorganization, rods of variable size, **intranuclear rods, cytoplasmic bodies**	This report
9 (M)	2d	2975 g (39 wGA)	c.686 T > C (p.Met229Thr) Exon 5	Hydramnios, lack of perception of fetal movement, hypotonia, facial weakness, respiratory distress, difficulties sucking & swallowing, multiple fractures	Yes	Deceased at 4d	Arthrogryposis, clubfeet	Fiber size variability, atrophy, endomysial fibrosis, cytoplasmic & intranuclear rods, cytoplasmic bodies	Myofibrillar disorganization, cytoplasmic rods of variable size emanating from enlarged Z-line segments, **intranuclear rods, cytoplasmic bodies, enlarged perinuclear space**	This report
10 (F)	15d	2100 g (34 wGA)	c.1132 T > C (p.Ter378GlnextTer47) Exon 7	Hypotonia, facial weakness	Yes	Alive at 13 y, permanent respiratory assistance, never walked	Arthrogryposis, hip retractions, pectus excavatum, left clubfoot, elongated face	Fiber size variability, type I fiber atrophy, endomysial fibrosis	Myofibrillar disorganization, cytoplasmic mini-rods	This report
11 (M)	1 m + 7 m	1900 g (33 wGA)	c.121C > T (p.Arg41*) Exon 2	Hydramnios, hypotonia, facial weakness, no respiration	Yes	Deceased at 22 m	-	Fiber size variability, type I fiber predominance, cytoplasmic mini-rods	Myofibrillar disorganization, mini-rods emanating from enlarged Z-line segments, **enlarged perinuclear space, abnormal NMJ,** prominent heterochromatin	Sparrow et al. 2003; Nowak et al. 2007
12 (M)	12d	Nd (33 wGA)	c.418G > C (p.Ala140Pro) (*dn*) Exon 3	Hydramnios, hypotonia, facial weakness, no respiration	Yes	Deceased at 5y	Scoliosis	Fiber size variability, cytoplasmic & intranuclear rods	Myofibrillar disorganization, cytoplasmic mini-rods, **large square-cut intranuclear rods**	Sparrow et al. 2003
13 (F)	20d + 2y6 m + 6y	2490 g (38 wGA)	c.466G > C (p.Asp156His) Exon 4	Hypotonia, respiratory distress	Yes	Deceased at 9y	Severe cardiomyopathy from early life and died of heart end-stage failure (while tracheostomized and able to walk)	Fiber size variability, endomysial fibrosis, cap-like filament aggregates, cytoplasmic rods	Thin filament accumulations, mini-rods and elongated rod within large filamentary areas	Lornage et al. 2020

*Nd *not determined; *d *days; *w* weeks; *m* months; *y *years; *wGA *weeks’ gestational age; *NMJ *neuromuscular junctions

### Muscle biopsies—histology, ultrastructure, immunofluorescence

All 13 patients underwent open muscle biopsy of the deltoid or quadriceps muscles. Patients 5, 11, and 13 underwent multiple biopsies. For morphological analyses, transverse 10 µm snap frozen cryostat sections were stained with standardized histological and histoenzymological techniques [38] and digital photographs were obtained with the AxioCam HRc camera (Zeiss, Oberkochen, Germany). Cytoplasmic/sarcoplasmic rods, intranuclear rods, and cytoplasmic bodies were assessed on Gomori trichrome-stained sections.

Electron microscopy studies were performed on muscle biopsy specimens from all 13 patients by standardized techniques [19]. The grids were viewed on a CM120 (Philips, Amsterdam, The Netherlands) and a Met Jeol 1400 Flash electron microscope (Jeol, Tokyo, Japan). Immunofluorescence studies were carried out on 8 µm muscle sections from the four patients with sufficient biological material and two age-matched controls. Images were acquired on a Ti2 microscope (Nikon, Tokyo, Japan) driven by Metamorph (Molecular Devices, San José, USA), and equipped with a CSU-W1 spinning disk head (Yokogawa, Tokyo, Japan) coupled with a Prime 95 sCMOS camera (Photometrics, Tucson, USA) and a 100 × oil-immersion objective lens. Super-resolution images were obtained using the LiveSR module (Gataca Systems, Massy, France). The following primary and secondary antibodies were used:

**Table:** 

Primary antibodies	Epitope	Origin	Dilution	Manufacturer
MAB 414 (ab24609)	Nuclear pore complex	Monoclonal (mouse)	1:100	Abcam
MANEM 5 (8A1)	Emerin	Monoclonal (mouse)	1:100	Glenn Morris
MANEM 8 (7B9)	Emerin	Monoclonal (mouse)	1:100	Glenn Morris
MANLAC 1 (4A7)	Lamin A/C	Monoclonal (mouse)	1:100	Glenn Morris
MANNES 1A (7A12)	Nesprin 1	Monoclonal (mouse)	1:100	Glenn Morris
MANNES 1E (8C3)	Nesprin 1	Monoclonal (mouse)	1:100	Glenn Morris
MANNES 2C (12A5)	Nesprin 2	Monoclonal (mouse)	1:100	Glenn Morris
MANNES 2E (18F7)	Nesprin 2	Monoclonal (mouse)	1:100	Glenn Morris
MANNES 2F (11C5)	Nesprin 2	Monoclonal (mouse)	1:100	Glenn Morris
A9357	Cardiac actin	Monoclonal (mouse)	1:500	Sigma Aldrich
L9393	Laminin	Polyclonal (rabbit)	1:50	Sigma Aldrich
**Secondary antibodies**	**Reference**	**Specificity**		**Manufacturer**
ALEXA Fluor 555	A-21422	Anti-mouse (red)	1:300	Life technologies
ALEXA Fluor 488	A-11034	Anti-rabbit (green)	1:300	Life technologies
DAPI	D-1306	Nuclei (blue)	1:1000	Life technologies

The A9357 anti-cardiac actin antibody has been used according the manufacturer’s recommendations to ensure specificity.

### Genetics—exome and Sanger sequencing

DNA was extracted from peripheral blood by routine methods. Exome sequencing was carried out for families 3, 4, 5, and 9, panel sequencing covering 210 neuromuscular genes (MYOdiagHTS) for families 1 and 7, and direct Sanger sequencing of all coding *ACTA1* exons and adjacent splice-relevant regions for families 2, 6, 8, and 10. For exome sequencing, leukocyte DNA libraries were prepared with the SureSelect Human all Exon 50 Mb capture library v5 (Agilent, Santa Clara, USA) and paired-end sequenced on an Illumina HiSeq2500 (Illumina, San Diego, USA). Sequence data were aligned to the GRCh37/hg19 reference genome, and variants were filtered and ranked based on their frequency in the internal and gnomAD databases, their predicted impact and segregation, and their expression pattern and known implication in human disorders. The presence and segregation of the variants was confirmed by Sanger sequencing. The *ACTA1* mutations were numbered according to GenBank NM_001100.4 and NP_001091.1.

## Results

### Clinical data

The ten novel patients constituting our cohort are sporadic cases from unrelated families without ancestral history of a neuromuscular disorder. All presented with antenatal or neonatal onset of a severe myopathy. The clinical, genetic, and histological features are summarized in Table 1, and compared to three previously reported patients with analogous phenotype [18, 24, 36].

Antenatal signs including hydramnios and reduced foetal movements were noted for patients 5 and 9, and patients 2 and 10 were born prematurely with a low birth weight. All neonates presented with pronounced hypotonia and hypomobility at birth requiring immediate mechanical ventilation followed by tracheostomy. Sucking and swallowing difficulties partially associated with hyper-salivation were seen in most infants and necessitated tube feeding and subsequent gastrostomy. Additional clinical features encompassed arthrogryposis (7$\times$), facial anomalies (3$\times$), high-arched palate (3$\times$), and skeletal deformities including clubfoot/clubfeet (4$\times$), pectus excavatum (2$\times$), scoliosis (1$\times$), and short ribs (1$\times$). Major hip retractions with legs bent over the abdomen at birth were observed in patients 8 and 10. None of the patients manifested an involvement of extraocular muscles.

The disease course was severe and most often fatal. Five neonates deceased during the first weeks of life (patients 1, 2, 4, 6, and 9), and two infants before the age of three years (patients 5, 7) (Table1). The surviving children are 4 and 13 years of age (patients 3 and 10), use orthopaedic corsets, are under permanent respiratory assistance, and never acquired walking. The oldest patient can move his hands, is able to write, and attends regular school courses.

### Identification of *ACTA1* mutations in all patients

**Fig. 1:**
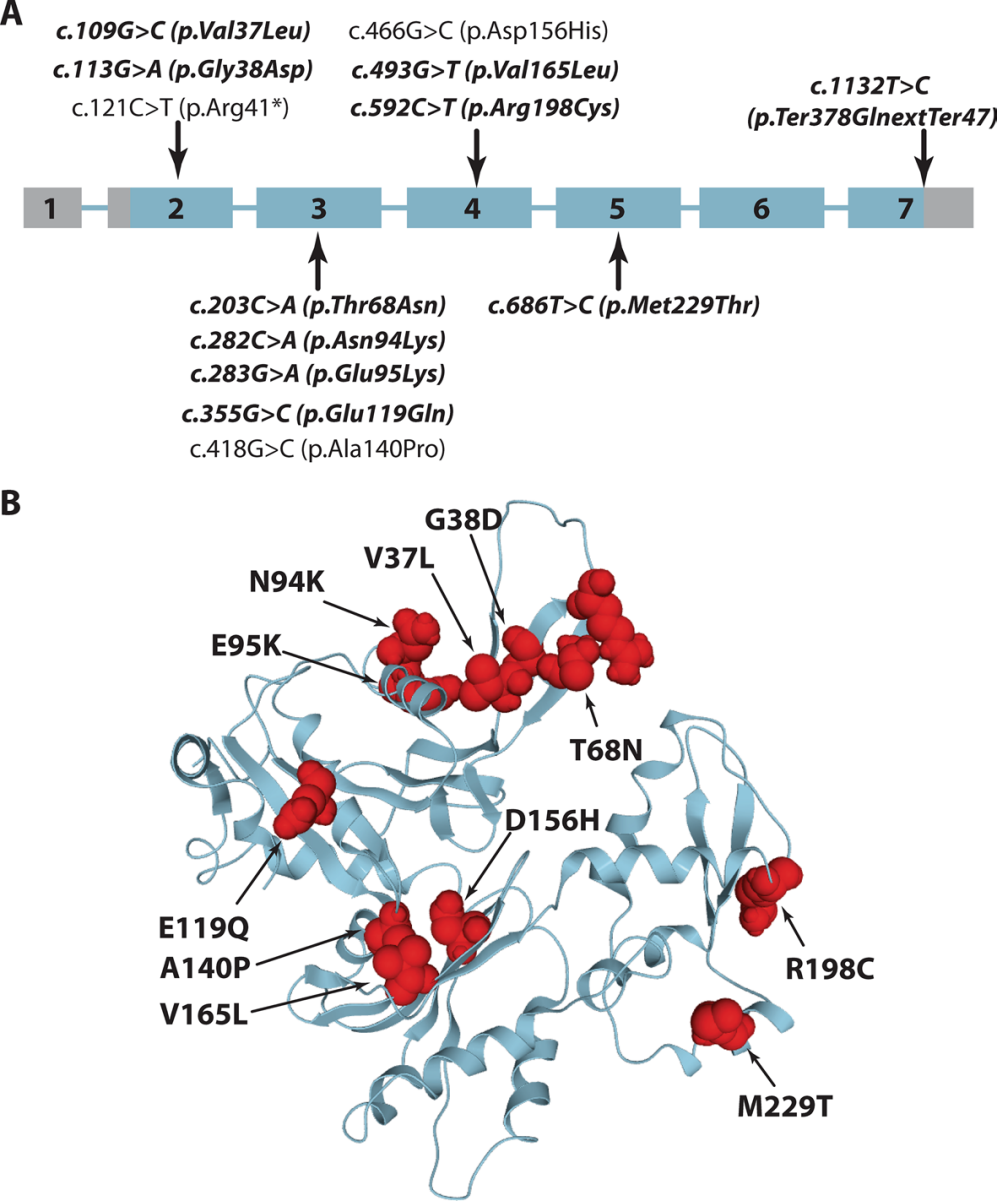
Overview of the *ACTA1* mutations. **A** Schematic representation of the seven *ACTA1* exons and position of the mutations in the ten novel patients (bold) and in the previously reported cases (light). **B** Resolved 3D protein structure of an αskm-actin monomer with position of the missense mutations highlighted in red

Exome, panel, or Sanger sequencing was carried out for the ten patients and the available clinically unaffected parents and uncovered *ACTA1* mutations in all families. These encompassed heterozygous missense mutations affecting highly conserved amino acids in patient 1 (c.109G > C; p.Val37Leu), patient 2 (c.113G > A; p.Gly38Asp), patient 3 (c.203C > A; p.Thr68Asn), patient 4 (c.282C > A; p.Asn94Lys), patient 5 (c.283G > A; p.Glu95Lys), patient 6 (c.355G > C; p.Glu119Gln), patient 7 (c.493G > T; p.Val165Leu), patient 8 (c.592C > T; p.Arg198Cys), and patient 9 (c.686 T > C; p.Met229Thr), as well as a stop-loss mutation predicted to generate an elongated protein with 47 supernumerary amino acids in patient 10 (c.1132 T > C; p.Ter378GlnextTer47), (Table 1 and Fig. 1). The missense mutations were evenly distributed over exons 2, 3, 4, and 5, and had no predicted impact on splicing. None was listed in the gnomAD public human variant database (https://gnomad.broadinstitute.org/), but c.109G > C (p.Val37Leu; initially described as p.Val35Leu) [36], c.203C > A (p.Thr68Asn) [17], c.282C > A (p.Asn94Lys) [6], c.283G > A (p.Glu95Lys), c.493G > T (p.Val165Leu, initially described as p.Val163Leu) [14, 25, 36, 40], c.592C > T (p.Arg198Cys) [17], c.686 T > C (p.Met229Thr) [17, 36], and c.11132 T > C (p.Ter378GlnextTer47) [39] have previously been reported in unrelated NM cases. Furthermore, the LOVD mutation database lists the c.283G > A (p.Glu95Lys) mutation found in patient 5, and also registered different mutations affecting residues Val37, Gly38, Thr68, Asn94, Val165, Arg198, Met229, or the stop codon (https://databases.lovd.nl/shared/variants/ACTA1/unique).

Segregation analyses revealed a de novo occurrence of the mutations in families 1, 2, 3, 4, 5, 6, 9, and 10, while parental DNA samples were not available for family 8 (Table 1). The c.493G > T (p.Val165Leu) missense in patient 7 was also found in the leucocyte DNA from the clinically unaffected mother with a low signal intensity on the electropherogram, indicating a mosaic pattern of the mutation.

Taken together, all *ACTA1* mutations described in the present study either arose de novo or were described in previously reported nemaline myopathy cases, pointing out their causality and pathogenicity.

### Common histopathological and ultrastructural hallmarks

**Fig. 2:**
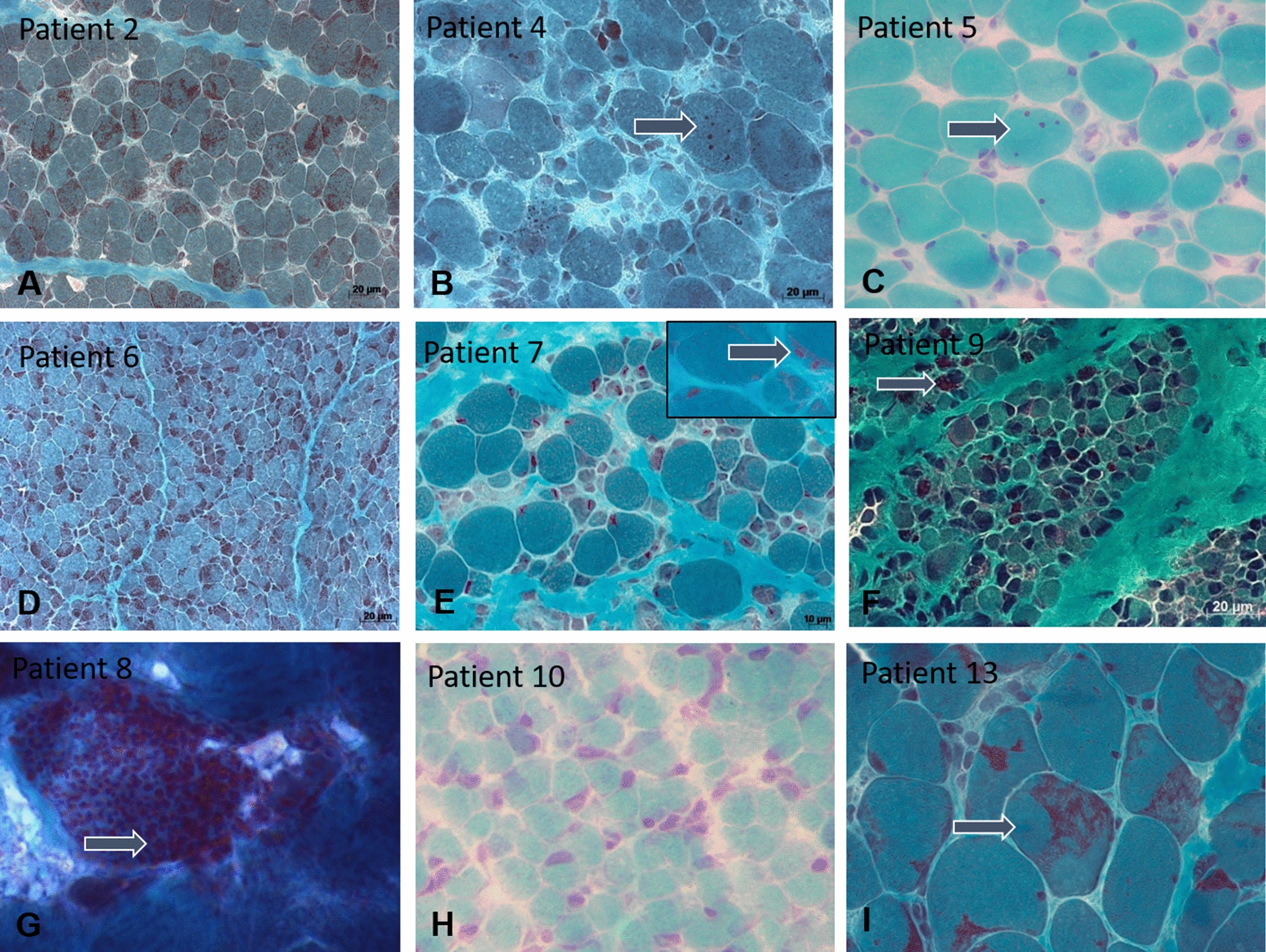
Muscle histology. Gomori trichrome staining on transverse muscle sections evidenced fibre size variability throughout the samples. The images also show an increased interstitial connective tissue in patients 4, 5, 7, 10, and 13 (**B**, **C**, **E**, **H**, **I**), abundant cytoplasmic rods in patients 2, 6, 8, 9, and 13 (**A**, **D**, **F**, **G**, **I**), cytoplasmic bodies (arrows) in patients 4, 5, 8, and 9 (**B**, **C**, **F**, **G**), major accumulations of thin filaments in patient 13 (**I**), as well as intranuclear rods in patient 7 (arrow, highlighted in the inset). In patient 10 (**H**), no rods or cytoplasmic bodies were observed

The ten novel patients enrolled in the study underwent open muscle biopsies between 2 days and 3 months of life, and patient 5 had an additional biopsy at a later stage (Table 1). The muscle sections were examined through a standard panel of histological and histochemical stains and revealed distinct structural anomalies and pathological protein accumulations. All muscle biopsy specimens displayed prominent fibre size variability (Fig. 2) associated with type I fibre atrophy, often accompanied by type I fibre predominance and occasionally by endomysial fibrosis. We also detected numerous fibres containing scattered central and clustered subsarcolemmal rods in patients 1, 2, 3, 6, 8, and 9, intranuclear rods in patients 6, 7, 8, and 9, and cytoplasmic bodies in patients 4, 5, 8, and 9. Signs of necrosis and muscle fibre degeneration were not seen. In patients 4, 5, 7, and 10, no cytoplasmic rods were detectable by light microscopy.

**Fig. 3:**
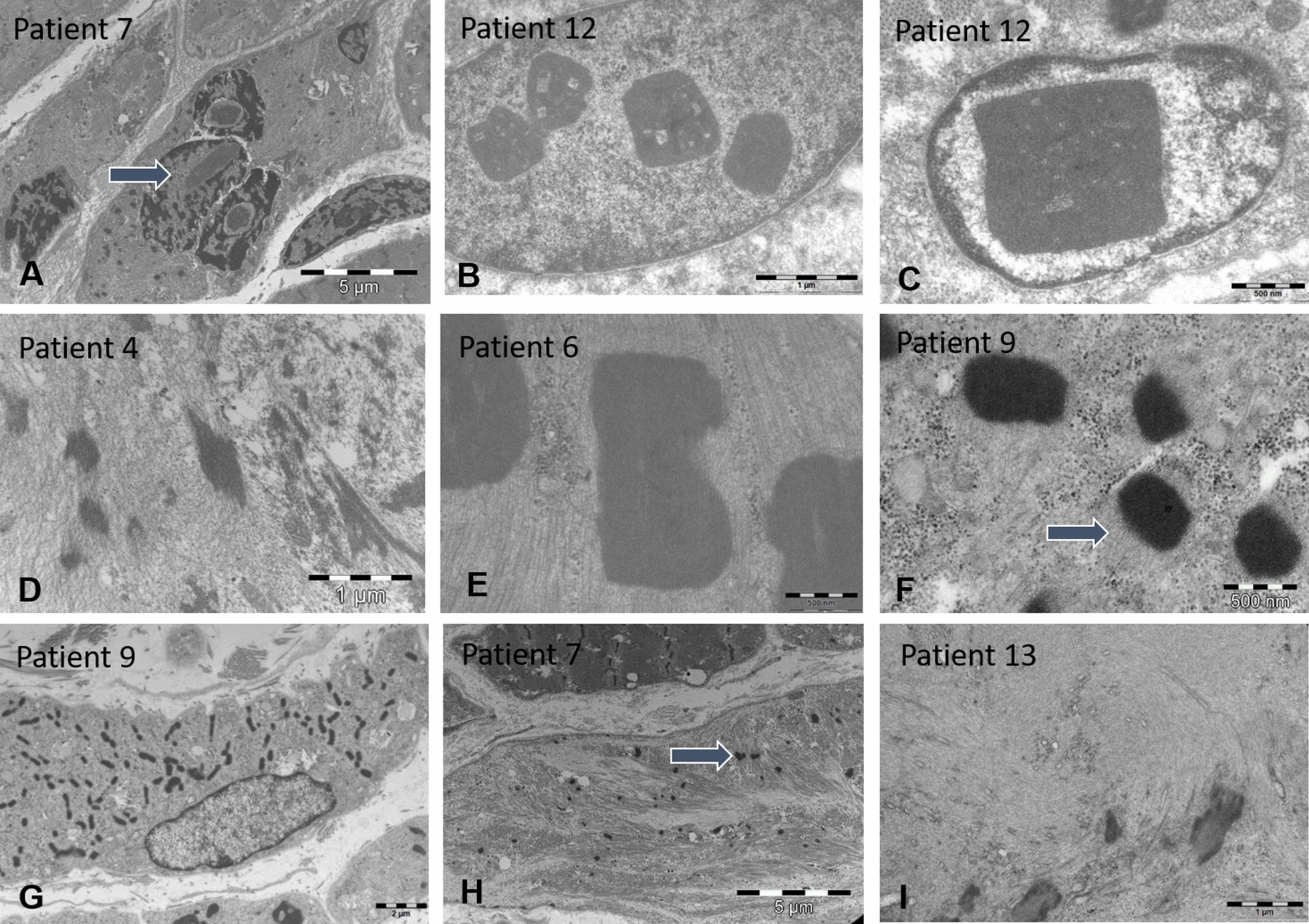
Cytoplasmic and intranuclear rods by electron microscopy. Ultrastructural investigations of the muscle biopsy specimens illustrate cytoplasmic rods with the typical lattice structure in patients 4, 6, and 9 (**D**, **E**, **F**), thin filaments emanating from thickened Z-lines in patients 6 and 9 (arrow) (**E**, **F**), numerous intranuclear rods in patients 7 (arrow) and 12 (**A**, **B**, **C**), accumulations of thin filaments in patients 7 (arrow) and 13 (**H**, **I**), and major myofibre disorganization in patients 4, 7, and 9 (**D**, **G**, **H**). Note the square shape of the intranuclear rods in patient 12 (**B**, **C**)

**Fig. 4:**
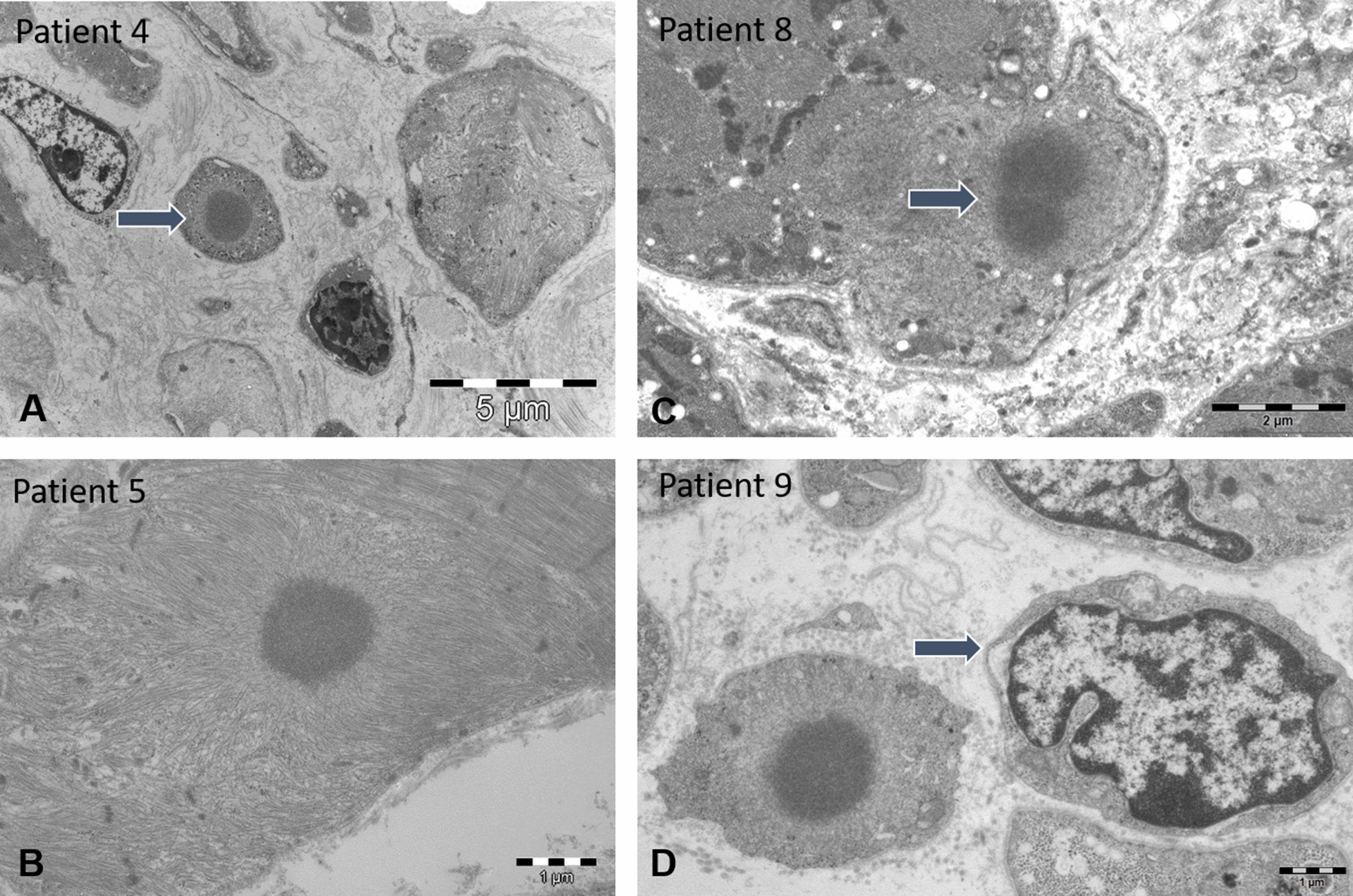
Cytoplasmic bodies by electron microscopy. Cytoplasmic bodies showing the characteristic halo of radiating filaments as illustrated on muscle sections from patients 4, 5, 8, and 9 (arrows, **A**–**D**). The patients also displayed fibre size variability, myofibre disorganization and endomysial fibrosis. The enlargement of the perinuclear space is indicated in patient 9 (arrow, **D**)

**Fig. 5:**
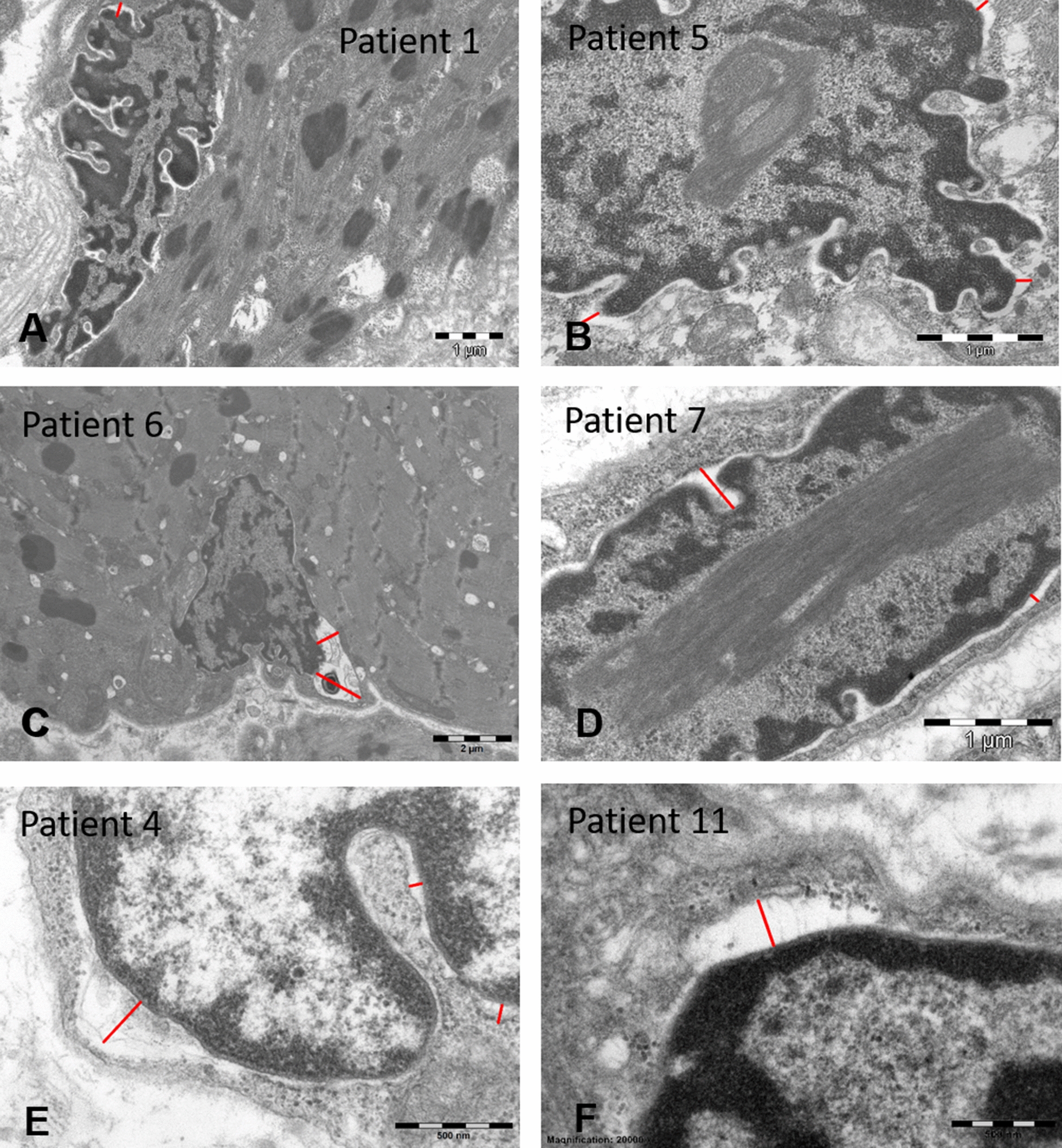
Ultrastructure of the nuclei. Electron microscopy detected nuclear anomalies in the muscle samples including intranuclear rods in patients 5 and 7 (**B**, **C**), and especially a significant enlargement of the perinuclear space scaling up to 165 nm in patient 5 (**B**), 195 nm in patient 1 (**A**), 217 nm in patient 4 (**E**), 230 nm in patient 11 (**F**), 390 nm in patient 7 (**D**), and 1230 nm in patient 6 (**C**) as indicated by the red bars. Patient 1 additionally displayed dense heterochromatic areas (**A**)

**Fig. 6:**
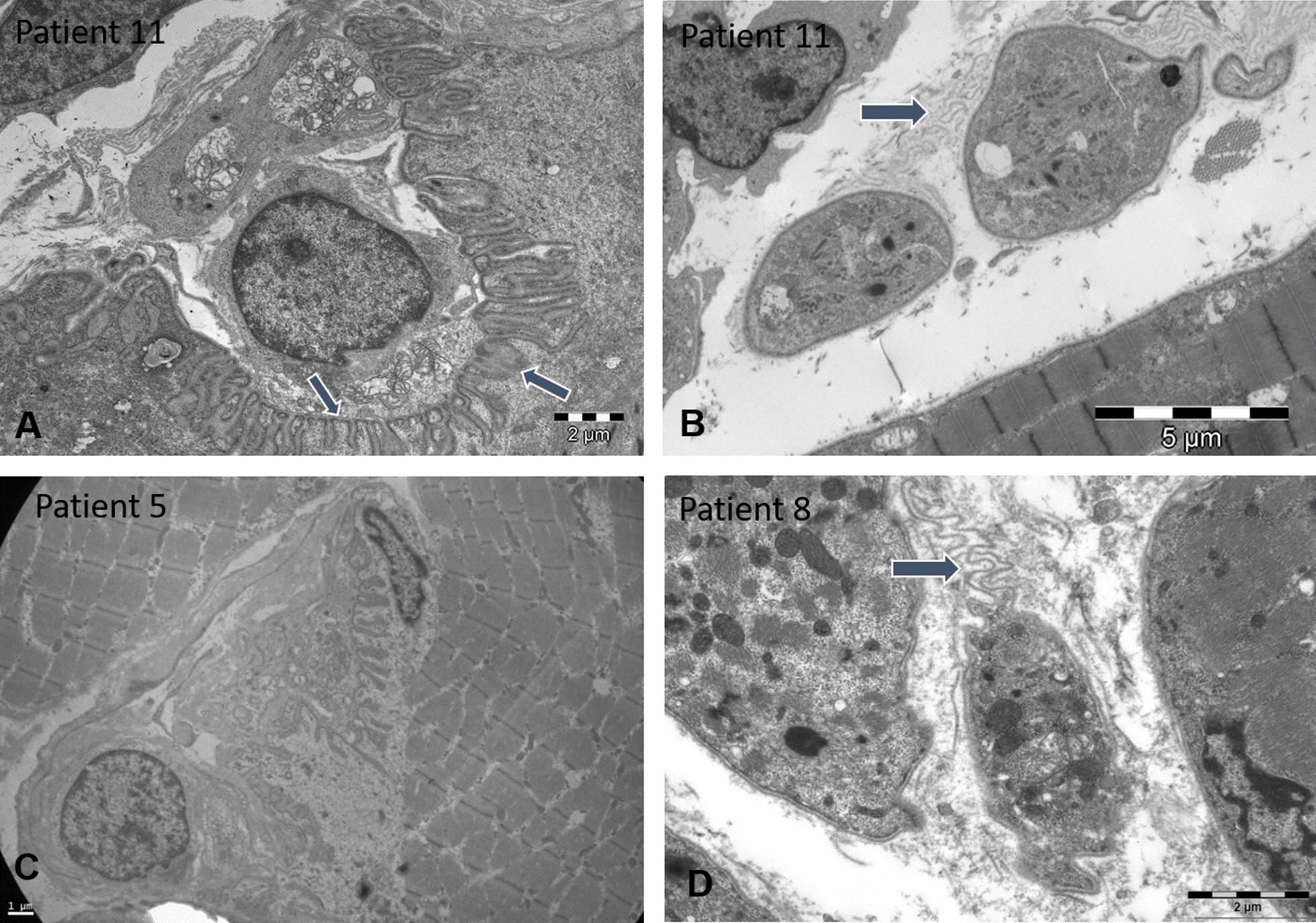
Ultrastructural endplates and membrane anomalies. Electron microscopy uncovered an abnormal organization of the skeletal muscle basal membrane in atrophic fibres showing ripples of excessive membrane projecting into the interstitium in patients 8 and 11 (arrows, **B**, **D**), and depleted postsynaptic membrane fold of the neuromuscular junctions in patients 5 and 11 (**A**, **C**, arrows)

Ultrastructural analyses of the muscle samples by electron microscopy confirmed the presence of cytoplasmic rods of variable size and shape, sometimes with thin filamentous protrusions as in patients 2, 6, and 7, or emanating from thickened Z-line segments as in patients 1 and 9 (Fig. 3). We also found mini-rods in patients 1, 4, 5, 7, and 10, and intranuclear rods in patients 5, 6, 7, 8, and 9 (Figs. 3 and 5). Cytoplasmic bodies characterized by the typical halo of radiating filamentous material were observed in patients 2, 4, 5, 8, and 9 (Fig. 4). Apart from these histopathological features classically characterizing nemaline myopathy, we also detected structural myofibre anomalies affecting the nuclear envelope, the nuclear lamina, and the neuromuscular junction (Figs. 5 and 6). Indeed, on muscle sections from patients 1, 4, 5, 6, 7, and 9, the perinuclear space appeared significantly enlarged in 10 – 12% of the fibres. In contrast to the average distance of 40 nm between the inner and the outer nuclear membrane in control myofibres, the width of the perinuclear space scaled up to 1200 nm in our patients (Fig. 5). Patient 1 additionally displayed increased spots of dense heterochromatin. Myofibrillar disorganization was prominent throughout the muscle biopsy specimens, and many atrophic fibres showed an undulating and partially detached basal lamina projecting into the interstitial tissue (Figs. 4 and 6). We also noted that all neuromuscular junctions were abnormal in patient 5, manifesting a reduced number of postsynaptic membrane folds and impoverished sub-neural structures (Fig. 6).

Taken together, the patients of our cohort presenting with severe *ACTA1*-related nemaline myopathy manifested major structural anomalies on the muscle biopsy specimens including a high frequency of cytoplasmic bodies and intranuclear rods, an unusual enlargement of the perinuclear space, and—in a single case—an aberrant architecture of the neuromuscular junction.

### Comparison with other severe *ACTA1* patients and immunohistopathological analysis of nuclei

Nuclear envelope deformities in combination with rods and cytoplasmic bodies constituted the principal anomalies on the muscle biopsy specimens from our patients. In order to conclude on the potential relevance of these structural aberrations as disease signature of severe *ACTA1*-related nemaline myopathy, we reviewed the clinical data from 3 previously reported cases with similar clinical presentation [18, 24, 36], and performed complementary investigations on the muscle biopsy specimens. Patient 11 harboured a homozygous nonsense mutation, and patients 12 and 13 carried heterozygous missense mutations (Fig. 1). Analogous to our ten unreported patients described here, the three additional cases presented with severe neonatal hypotonia and respiratory distress, and all deceased in infancy or childhood (Table 1). Thorough morphological analyses of the muscle sections revealed the same set of principal architectural aberrations including fibre size variability, atrophy associated with endomysial fibrosis, cytoplasmic and intranuclear rods, cytoplasmic bodies, and especially the enlargement of the perinuclear space (Figs. 3–5). Moreover, all neuromuscular junctions showed a reduced number of postsynaptic membrane folds and impoverished sub-neural structures in patient 11 (Fig. 6), and patient 12 displayed a large number of intranuclear-rods with unusual square shape (Fig. 3).

**Fig. 7:**
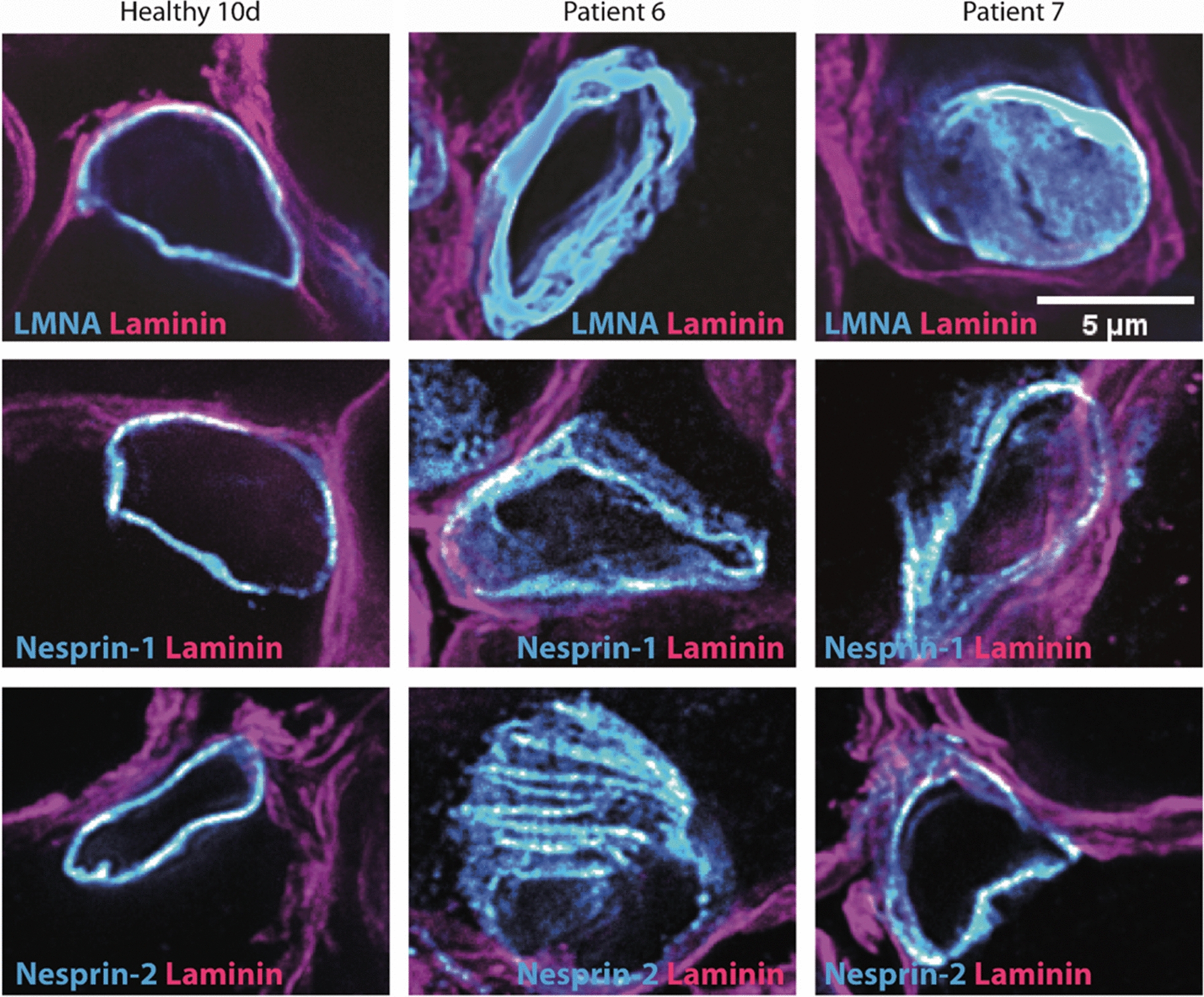
Immunofluorescence of nuclei. Representative images of muscle samples showing an abnormal nuclear shape in patients 6 and 7 compared with the age-matched control as illustrated by the lamin A/C, Nesprin-1, and Nesprin-2 signals. Laminin indicates the basal lamina. The images correspond to a single z-plane after super-resolution of confocal acquisition

To shed light on the factors contributing to the pathologic widening of the perinuclear space in our patients, we investigated the localization of inner and outer nuclear membrane proteins forming the LINC (linker of nucleoskeleton and cytoskeleton) complex and connecting the nuclear lamina with the cytoskeleton. Immunofluorescence on muscle sections from patients 6 and 7 revealed abnormal lamin A/C, Nesprin-1, and Nesprin 2 signals compared with the control (Fig. 7 and Additional file 1: Fig. S1). Confocal microscopy across the nuclei and quantification of the signal intensities through line scans confirmed the narrow localization of the three proteins at the nuclear envelope in the control muscle samples, while the distribution pattern of lamin A/C, Nesprin-1 and Nesprin 2 was significantly wider in the *ACTA1* patients (Fig. 7 and Additional file 1: Fig. S1). Indeed, lamin A/C signals were detectable within the nuclei, and Nesprin-1 and Nesprin-2 signals in the perinuclear region in patients 6 and 7, indicating an impairment of the NE integrity.

### Increased cardiac α-actin expression in the patients with longer lifespan

**Fig. 8:**
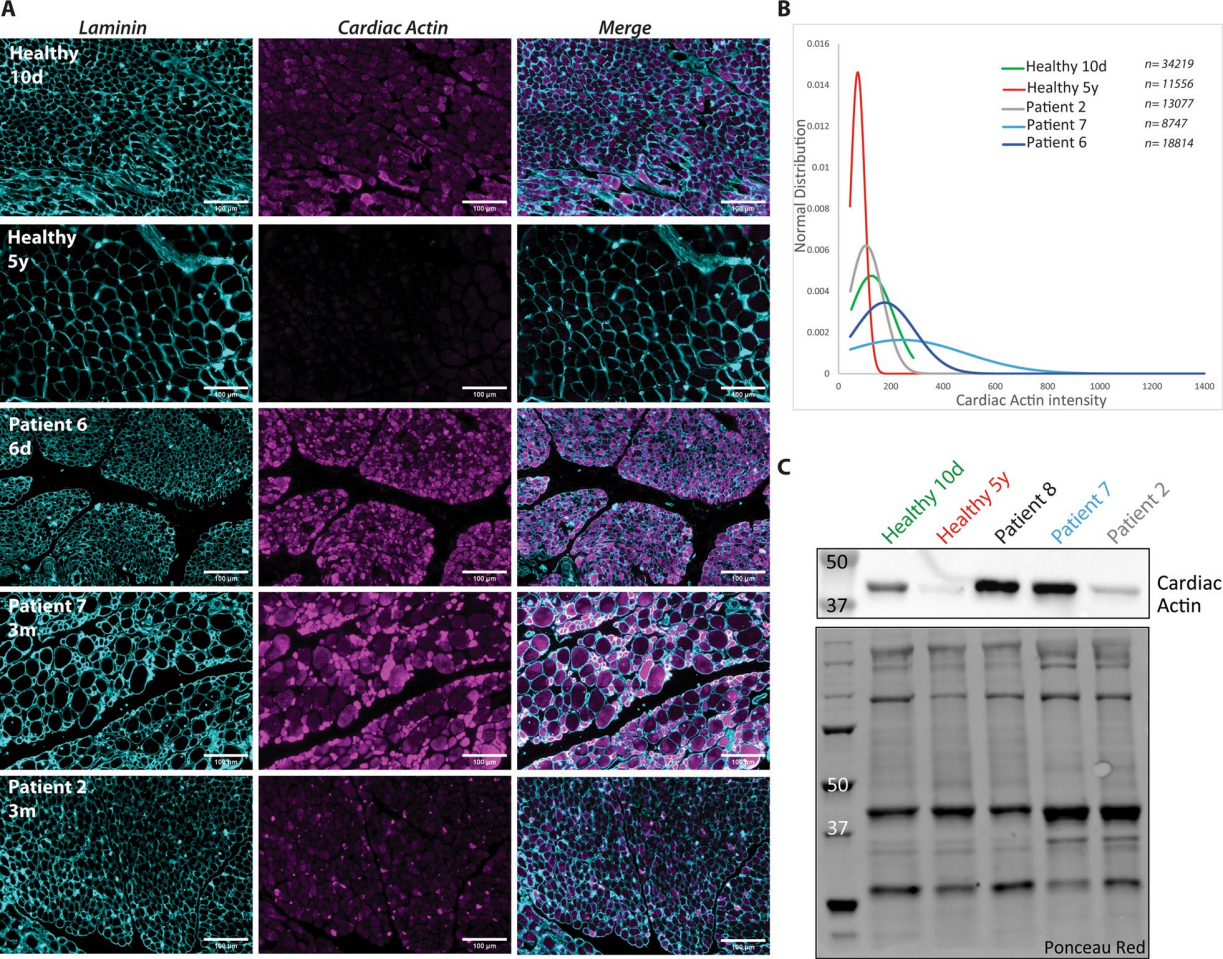
Cardiac α-actin immunofluorescence. (**A**) Cardiac α-actin signals (Magenta) on muscle sections from patients and age-matched controls. Laminin appears in Cyan. Scale bar:100 $\upmu$m (**B**) Normal distribution of each fibre intensity for cardiac actin demonstrates an increased cardiac α-actin level in patients 6 and 7 compared with the other patients and the controls. The number of analysed fibres is indicated for each patient. (**C**) Western blot on muscle extracts shows intense cardiac α-actin bands in patients 7 and 8, and a lower expression in patient 2 and in the controls. Ponceau red served as loading control

The cohort described here is composed of patients with severe nemaline myopathy. While most deceased within the first days or weeks of life, others lived into childhood or early adolescence. To investigate the potential molecular causes accounting for the intrafamilial variability, we determined the expression level of cardiac α-actin by western blot and immunofluorescence on muscle samples from our patients and age-matched healthy controls (Fig. 8). Cardiac α-actin differs from αskm-actin in only 4 amino acids, is the predominant α-actin form in skeletal muscle during embryonic development, and is replaced by αskm-actin around birth [35]. Patients with homozygous *ACTA1* null mutations and a total absence of αskm-actin in skeletal muscle were found to persistently express cardiac α-actin after birth, which may have contributed to a less severe disease course in the reported cases [24]. In agreement with its physiological role in embryonic muscle development, cardiac α-actin was detectable on muscle samples from the younger control of 10 days, and was largely absent in the older control of 5 years (Fig. 8). Of note, the cardiac α-actin signals were significantly enhanced in muscle samples from the patients with longer lifespan (patients 7 and 8, lived until 2.5 and 5 years, respectively) compared with the patients perished in the neonatal period (patients 2 and 6, deceased at 1 and 3 months, respectively), suggesting a compensatory effect of cardiac α-actin in patients with *ACTA1* missense mutations.

## Discussion

Here, we describe ten novel patients with severe nemaline myopathy associated with dominant *ACTA1* mutations. Through in-depth histological, ultrastructural, and immunofluorescence investigations of the ten new and three published cases, we expand the spectrum of morphological muscle anomalies in severe *ACTA1*-related NM, and we also provide a potential disease signature encompassing intranuclear rods, cytoplasmic bodies, neuromuscular junction abnormalities, and the enlargement of the perinuclear space.

### The genetics of severe *ACTA1*-related NM

To date, more than 200 *ACTA1* mutations have been described, and they give rise to a spectrum of muscle disorders collectively classified as actinopathies and varying in disease severity and the presence of histopathological hallmarks on muscle biopsy specimens [17, 23] (https://databases.lovd.nl/shared/genes/ACTA1). Missense mutations are most commonly found, but nonsense mutations, splice mutations, as well as insertions/deletions have also been described. The mutations are evenly distributed over the six coding exons of *ACTA1*, and the absence of an obvious hotspot is supported by the observation that the number of reported mutations correlates with the size of the individual exons [17]. Accordingly, nine of our novel patients carried *ACTA1* missense mutations, and they were spread along the exons.

*ACTA1* is a highly conserved gene barely tolerating genetic modifications. Any mutation is likely to have major pathogenic effects on αskm-actin function and muscle contraction, which presumably accounts for the elevated number of *ACTA1* patients with severe clinical presentation. Consistently, all our patients manifested marked disease signs in the neonatal period with pronounced hypotonia, general muscle weakness, and respiratory distress. In agreement with the high degree of premature lethality of affected individuals, only *ACTA1* mutations associated with a milder phenotype have been reported with autosomal dominant inheritance, while most arise de novo [17]. Indeed, nine of the ten mutations described here were undetectable in the parents. For one family, parental DNA was not available for segregation studies, but the index patient had no ancestral history of a muscle disorder, suggesting that the respective mutation occurred de novo. The c.493G > T (p.Val165Leu) missense mutation in patient 7 constitutes an exception. Exome sequencing detected the same nucleotide transversion with a low number of reads in the leucocyte DNA from the clinically unaffected mother, indicating mutation mosaicism. Another *ACTA1* mosaic mutation associated with marked left–right asymmetry has recently been reported [18]. The c.1132 T > C mutation found in patient 10 affects the terminal stop codon of *ACTA1* and is predicted to result in an elongated αskm-actin protein. It remains to be determined how and to what extent the 47 supernumerary amino acids interfere with αskm-actin structure, function, and the interaction with other proteins, but the fact that the very same mutation has been described in unrelated cases with severe disease presentation [39] is in favour of a strong pathogenic effect. However, our patient is now 13, while the published cases deceased in infancy, suggesting that additional genetic and non-genetic factors modulate disease development and survival.

### Intranuclear rods, cytoplasmic bodies, and an enlarged perinuclear space as histopathological hallmarks of severe NM

The pathognomonic nemaline rods are the most common histopathological feature in NM patients [23, 30]. They are found in the cytoplasm throughout most muscle biopsy specimens from affected individuals, irrespectively of the implicated gene and the causative mutation [14]. The rods are essentially composed of thin filaments and Z-line material forming the lateral boundaries of the contractile units, and contain α-actinin, actin, tropomyosin, myotilin, γ-filamin, cofilin-2, telethonin, and nebulin [34]. Noteworthy, cytoplasmic rods were invisible by histology in five of our patients, and were ultimately detected through ultrastructural analyses by electron microscopy. This is possibly due to the restricted resolution of light microscopy and the little size of the rods, but may also reflect the absence of rods on specific muscle sections or is associated with the age of the patient at the time of muscle biopsy. Indeed, myofibres from neonates are often small, and it may be challenging to detect histological lesions within hypotrophic fibres. Patient 13 underwent muscle biopsies at 20 days, 2.5 years, and 6 years, and we observed a distinct evolution of the histopathological features with barely detectable mini-rods on the first muscle biopsy specimen, and prominent clusters of rods with aggregation of thin filaments on the last muscle biopsy specimen.

In contrast to cytoplasmic rods, intranuclear rods are seen less often, and it is currently not fully understood why and how the rods form within the myonuclei [5]. It is however well known that the intracellular localization of α-actin is not restricted to the cytoplasm or to a given tissue or organism. Indeed, nuclear α-actin has been observed across species and in many different cell types, indicating a role in transcriptional regulation [15]. It has also been demonstrated that specific chemical substances and an increased temperature can stimulate the formation of intranuclear rods in cultured cells [8, 41], suggesting that mechanical or another form of cellular stress may trigger the abnormal accumulation of αskm-actin bundles in *ACTA1* patients. Of note, intranuclear rods are mainly found in severe *ACTA1* cases, and especially in patients carrying missense mutations affecting amino acids 139 to 165, encompassing the hinge domain responsible for protein flexibility [17, 21]. In our cohort, intranuclear rods were noted in five patients. All carried missense mutations encoded by exons 3, 4, and 5, indicating that structural modification of the central part of αskm-actin may indeed promote the formation of intranuclear rods. However, mutations between amino acids 139 and 165 were only found in two patients, suggesting that the sensitive region may be larger than currently assumed.

In analogy to nemaline rods, cytoplasmic bodies are also believed to derive from Z-line material. Cytoplasmic bodies are not pathognomonic for a particular neuromuscular disease as they can be found in myofibrillar myopathies, reducing body myopathy, myotonic dystrophy, or periodic paralysis [6, 33]. They are however rarely seen in *ACTA1*-related NM, and the few described cases were invariably severe [6, 11, 16, 37]. Noteworthy, one of these patients carried the same c.282C > A (p.Asn94Lys) missense mutation as our patient 4, and the c.283G > A (p.Glu95Lys) mutation affecting the adjacent amino acid was detected in patient 5. Both patients displayed cytoplasmic bodies on the muscle biopsy specimens, indicating a common role of αskm-actin residues 94 and 95 in the formation of cytoplasmic bodies.

Another striking histopathological feature in the muscle fibres of our patients is the abnormal nuclear shape. The nuclear envelope (NE) separates the nucleus from the cytoplasm in all eukaryotic cells and is composed of an outer and inner nuclear membrane bordering a perinuclear space of 30 to 50 nm [10]. The spatial and structural integrity of the nucleus is maintained by the LINC complex, which also controls nuclear orientation, and possibly plays a regulatory role in replication, DNA repair, and cell division [3]. The mechanical link between the nuclear envelope and the cytoskeleton is mediated by F-actin, which is essentially composed of cytoskeletal actins encoded by *ACTB* and *ACTG1* [27]. In our patients, inner and outer membranes were disassembled with an enlargement of the perinuclear space of up to 1200 nm and more, and consistently, we observed an abnormal localization of the LINC proteins Nesprin-1 and Nesprin-2. Together with the concurrent mislocalisation of lamin A/C, coating the inner nuclear membrane and implicated in transcriptional modulation [4], this suggests that the *ACTA1* mutations directly or indirectly impact on the function of F-actin as a molecular linker, and that the aberrant nuclear envelope architecture may interfere with gene expression as in nuclear envelopathies [2] and contribute to the severe phenotype of our patients. Of note, a single study previously reported irregularities in nuclear distribution and shape associated with chromatin abnormalities in both *ACTA1* and *NEB* patients [31], indicating that alterations of the nuclear envelope can also be seen in other nemaline myopathy forms and potentially involve a common pathomechanism. An enlargement of the perinuclear space has furthermore been reported in myogenic-type arthrogryposis multiplex congenita-3 (AMC3) patients harboring *SYNE1* mutations [1].

A significant loss of postsynaptic membrane folds was seen in one novel (Patient 5) and in one previously reported patient (Patient 11). However, biopsies are muscle fragments and usually reflect only a narrow tissue section. In most of our patients, the samples did not contain areas with neuromuscular junctions, precluding a general conclusion on the correlation between *ACTA1* mutations and aberrant NMJ architecture. However, this is clinically meaningful since motor, respiratory and bulbar fatigability is a relevant feature, often observed in nemaline myopathy. It is worth mentioning that NMJ anomalies are commonly seen in acquired and genetic myasthenic diseases (myasthenia gravis, congenital myasthenic syndromes (CMS)) and can also occur in congenital myopathies [28]. Treatment with salbutamol or with the cholinesterase inhibitor pyridostigmine is efficient in myasthenia gravis and in different CMS, and has been shown to improve muscle function in patients with *TPM3*-related congenital fibre type disproportion (CFTD) [22] and in patients with centronuclear myopathy (CNM) caused by *DNM2, MTM1*, or *RYR1* mutations [9, 12, 29], and may therefore suggest a therapeutic option for severe *ACTA1*-related NM.

### Potential genotype/phenotype correlation

The interfamilial disparity of disease course, severity, and lifespan in our patients probably reflects a combination of numerous individual parameters including the position of the mutation and pathogenic impact, ethnic origin, the diverging activity of modifier genes, and the intensity of clinical care. A genotype/phenotype correlation may nevertheless be drawn from the histological and ultrastructural anomalies on the muscle biopsy specimens. Indeed, none of the patients featuring at the same time intranuclear rods, cytoplasmic bodies, and perinuclear space enlargement survived beyond the age of 18 months, and from the patients living more than 3 years, all except one manifested none or only one of these hallmarks. This indicates that distinct missense mutations affecting specific amino acids or protein domains have a more deleterious effect on skeletal muscle integrity, and directly correlate with disease severity. However, it remains to be determined why and how other *ACTA1* missense mutations in the same region lead to a comparably milder phenotype. The relative expression of cardiac α-actin—although only assessed in four patients with sufficient biological material—appears to be another contributing factor. Indeed, patients 7 and 8 showed increased levels of cardiac α-actin in skeletal muscle, and both survived beyond the age of 2 years, while patient 2 and 6 expressed significantly less cardiac α-actin and lived for a few weeks only. In accordance, the overexpression of cardiac α-actin in *Acta1* KO mice was shown to preserve muscle function [26]. Overall, the findings in humans and mice suggest that cardiac α-actin is able to partially attenuate disease progression and to increase lifespan in *ACTA1*-related NM, and highlight the therapeutic potential of the cardiac actin paralogue.

## Concluding remarks

*ACTA1* is a major NM gene, and pathogenic mutations are most often associated with a severe clinical presentation and poor prognosis. Histological investigations of muscle biopsy specimens from affected neonates can be inconclusive in the absence of classical nemaline rods and may systematically require complementary ultrastructural analyses. *ACTA1* should be considered in patients with pronounced antenatal or neonatal muscle weakness, especially if electron microscopy on muscle sections detects intranuclear rods, cytoplasmic bodies, and an enlargement of the perinuclear space.

## Supplementary Information

**Additional file 1: Figure S1.** Myofibre nuclear envelope line scan profiles. Quantification of signal intensities at the middle of the nuclei shows an abnormal lamin A/C, Nesprin-1, and Nesprin-2 localization in patients 6 and 7 compared with the age-matched control. Red brackets show the extension of nuclear envelope proteins into the cytoplasm. Each graph corresponds to one nucleus. Bottom right: explanatory scheme of the analysis with the nucleoplasm depicted in grey.

## Data Availability

All data generated or analyzed during this study and concerning clinical and histological characteristics and *ACTA1* are included in this published article. Other DNA variants identified by panel or exome sequencing are not publicly accessible.
