# Supplementary figures and images for: Severe ACTA1-related nemaline myopathy: intranuclear rods, cytoplasmic bodies, and enlarged perinuclear space as characteristic pathological features on muscle biopsies
Source: Acta Neuropathol Commun. 2022 Jul 9;10:101. doi: 10.1186/s40478-022-01400-0 (PMC9271256; doi:10.1186/s40478-022-01400-0)

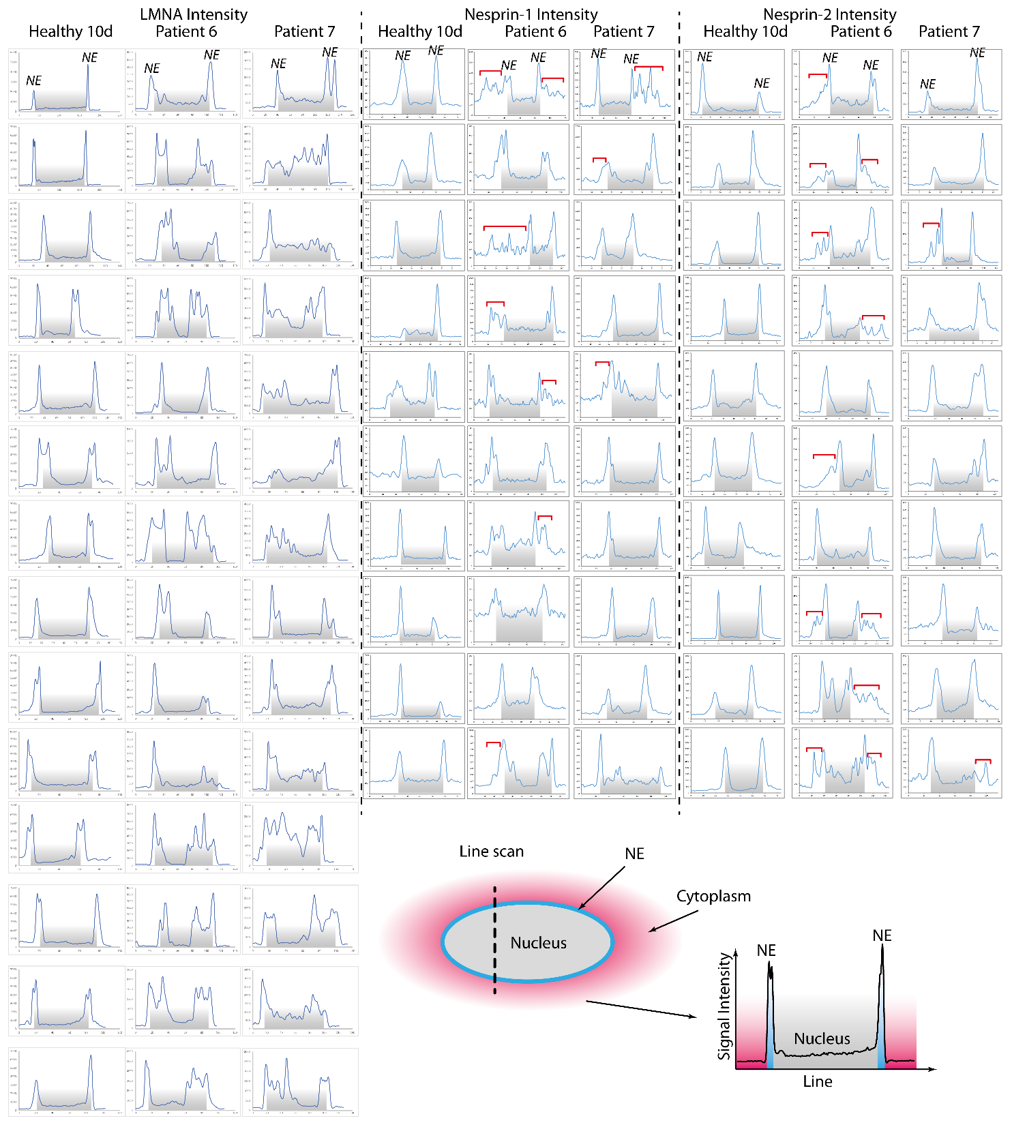

Supplement: Supplementary file 1 — Additional file 1: Figure S1. Myofibre nuclear envelope line scan profiles. Quantification of signal intensities at the middle of the nuclei shows an abnormal lamin A/C, Nesprin-1, and Nesprin-2 localization in patients 6 and 7 compared with the age-matched control. Red brackets show the extension of nuclear envelope proteins into the cytoplasm. Each graph corresponds to one nucleus. Bottom right: explanatory scheme of the analysis with the nucleoplasm depicted in grey. [file 40478_2022_1400_MOESM1_ESM.tif]
